# Effects of Different Rapid Weight Loss Strategies and Percentages on Performance-Related Parameters in Combat Sports: An Updated Systematic Review

**DOI:** 10.3390/ijerph20065158

**Published:** 2023-03-15

**Authors:** Luis Manuel Martínez-Aranda, Manuel Sanz-Matesanz, Guillermo Orozco-Durán, Francisco Tomás González-Fernández, Lorena Rodríguez-García, Amelia Guadalupe-Grau

**Affiliations:** 1 Physical Performance & Athletic Research Center, Faculty of Sports Sciences, University Pablo de Olavide, 41013 Seville, Spain; 2 MALab (Movement Analysis Laboratory for Sport and Health), Catholic University of Murcia, 30107 Murcia, Spain; 3 Faculty of Sport, Catholic University of Murcia, Guadalupe, 30107 Murcia, Spain; 4 Department of Physical Education and Sports, Faculty of Sport Sciences, University of Granada, 18071 Granada, Spain; 5 Department of Physical Activity and Sport Sciences, Pontifical University of Comillas, CESAG, 07013 Palma, Spain; 6 GENUD Toledo Research Group, Universidad Castilla-La Mancha, 45002 Toledo, Spain; 7 CIBER of Frailty and Healthy Aging (CIBERFES), Instituto de Salud Carlos III, ISCIII, 28220 Madrid, Spain

**Keywords:** recovery, weight loss, weigh-in, hydration, competitive performance, fight, striking sports

## Abstract

Most combat sports (CS) are structured in weight categories, and it is very common to carry out body weight adjustment strategies in order to compete in lower weight categories. For this reason, different rapid weight loss (RWL) strategies are usually performed to pass the pre-competition weigh-in test, and then a replenishment of fluids and carbohydrate-rich foods is conducted in an attempt to recover the weight and avoid a performance loss. In this context, no clear references have been found on whether these types of strategies have negative effects, impairing the athlete’s combat and/or physical performance. For this reason, the aim of this study was to review the scientific literature on the effect of rapid weight reduction strategies on the performance of CS athletes. A literature search was performed through four different databases (PubMed, SPORTDiscus, Web of Science and ScienceDirect). Four inclusion criteria were established as follows: (1) the subjects had to be competitors in the CS and carry out RWL strategies; (2) at least two measurement points, that is, normal conditions and dehydration condition; (3) measurements in a real competition or simulating the same conditions; (4) original research articles written in English or Spanish and available in full text. Finally, a total of 16 articles were finally included in this research. All subjects (*n* = 184) were athletes from combat disciplines, with a minimum of 3–4 years of practice, as well as with certain experience in RWL. Six of the studies reported that an RWL strategy of around 5% of body weight loss did not affect performance parameters. However, the other ten studies with RWL between 3 and 6% or even higher reported negative effects or impairments on different parameters related to performance and/or athlete’s psychophysiology, such as perceived fatigue, mood states, strength and power production, as well as changes in hormonal, blood and urine parameters, body composition, or the kinematics of the technical gesture. Although there is still no clear answer to the issue approached in this research, in general terms, it seems that in order to guarantee an acceptable athletic performance of the competitor, the weight loss should not exceed 3% to ≤5% of body weight together with ≥24 h for adequate (or at least partial) recovery and rehydration processes. In addition, it is highly recommended to lose weight progressively over several weeks, especially focusing on competitions lasting several days, as well as multiple rounds or qualifying stages.

## 1. Introduction

Combat sports (CS) are disciplines with different weight categories where it is common for athletes to compete in a category below their natural weight in order to gain a competitive advantage [1,2]. This advantage focuses on short-term rapid weight loss (RWL), coinciding with the official weigh-in day, when the competitor is placed in one category or another. This is followed by rehydration and carbohydrate intake to allow the athlete to gain weight and thus achieve an advantage over smaller competitors [3,4]. In fact, given the widespread use of this strategy in CS, nowadays it is practically considered a must in order to avoid a possible physical disadvantage due to size and/or strength [1,2] However, there is a great lack of knowledge regarding the ideal way to perform these weight reductions, often causing athletes to worsen their athletic performance in competition by performing protocols that are too extreme. As a common example, a very pronounced weight loss over an acceptable percentage in a very short period of time. Other strategies that could be considered extreme or unwise include an abusive calorie restriction in the days prior to the weigh-in, remaining hungry for long periods of time, using the sauna, or the use of pharmaceutical drugs on certain occasions that are often labelled as illegal by competition committees [5]. These methods can lead to severe dehydration, jeopardising not only purely sporting performance, but also the athletes’ physical health, especially as there is often no control or supervision during such a process [5,6,7].

Apart from the negative effects of RWL on parameters related to sports performance, such as strength (e.g., maximal handgrip and specific grip tests), power outputs, and specific endurance development [6], the cognitive and physiological consequences derived from these strategies must also be taken into consideration [8]. In terms of cognitive consequences, several studies showed that short-term memory and mood may be negatively affected, but once rehydration is conducted, these measures return to near baseline or normal values, so that these negative effects may be reversible [8]. In addition, a rapid weight loss may lead to a decrease in metacognitive performance, which may attenuate attention and anticipation skills, and thus affect decision-making processes [4,9].

On the other hand, the “weight cycling” (losing and regaining weight repeatedly) was shown to have marked adverse effects on the athlete’s physiology, e.g., in endocrine (low concentrations of serum testosterone and prolactin) or renal function. Concerning the renal function, decreases in the plasma volume could be noted, which contributes to a decrease in blood flow to the kidneys. This would therefore increase acidity, urine-specific gravity [6], and leakage of albumin and leucine amino peptidase into the urine (linked to ischaemia). Although the effect of weight cycling on strength or aerobic power performance is limited, it does significantly affect muscular endurance and cardiovascular system, considered essential in CS due to their particular competitive characteristics [2,6,7,10]. In addition, it is important to avoid specific carbohydrate restriction that may affect the athlete’s muscular capacity, favouring a more balanced calorie restriction in such weight loss strategies [10].

Most CS athletes consider hydration habits to be important for health and athletic performance, and even though they recognise negative effects on athletic performance due to RWL, they continue to engage in RWL [11]. Therefore, it is necessary to deepen the knowledge of RWL methodologies in order to clarify the existing claims thus far [12].

The most commonly used methods of RWL are based on a combination of active and passive sweating, fluid and food restriction, and the use of plastic or rubber clothing to induce profuse sweating [1,2,4,6,7] However, current studies show a new methodology that is gaining popularity in CS, the so-called “water loading”, purportedly decreasing body mass through an increasing of the urine production. That consists of consuming large volumes of water (7–10 L/d) for several days before a subsequent fluid restriction, which seems to be a safe and effective method of acute bowel evacuation [13]. This strategy could be interesting in lean athletes without much fat mass who are losing weight in order to weigh in for a specific category. At the same time and in parallel to the control of fluid intake, the most common methodologies of dietary restriction are the consumption of low-fibre foods, which with a correct intake strategy allow the athlete to better restore energy after weigh-in [3]. However, the influence of RWL on athletic performance is not entirely clear, although it seems that weight cuts greater than 5% of body weight would make it more difficult to repeat an effort continuously over time [12].

On the other hand, although existing studies investigating weight loss strategies in CS mainly focus on the study of athletes’ hydration index [14,15] or their anthropometric characteristics [3,16], it is essential to understand the influence of these strategies on athletic performance, as they are aimed precisely at gaining a competitive advantage [2].

More recently, Brechney et al. [1] focused their review with a meta-analysis on the influence of RWL strategies on performance (tests focused on the strength, power, and endurance of athletes), and the recovery process in CS. In this study, small to moderate reductions in overall exercise performance, maximal strength, and repeated high intensity effort were found between pre- and post-RWL strategy (*p* < 0.03). However, they also indicated that the entire weight cutting process (RWL to rapid weight gain) is not sufficient to change the values of an overall exercise performance, as well as short-term tests applied to CS (anaerobic capacity, maximal strength, and so on). Even though they provided very valuable information on the influence of RWL protocols on performance, they did not include as a criterion the application of strategies in real or simulated competitive situations, with almost all the studies including isolated laboratory tests. Actually, the studies considering data collected immediately post-competition or -bout were excluded in their analysis. Therefore, their practical application to the competition or tournament period, as well as the interest of coaches and athletes, could be limited to a certain extent.

Based on this lack of specific knowledge in competitive situations, the present study focuses on the effect of different RWL strategies on athletic-performance-related parameters of CS (power, strength, jump ability, speed, specific combat exercise…, among others) in real or simulated competitive environments. By understanding the impact of those RWL strategies on CS athletes’ health and performance, coaches will gain useful information on what strategies to avoid prior to competitions, and how training processes can be optimised in the run-up to competitions.

## 2. Materials and Methods

### 2.1. Search Strategy

This systematic review was conducted in accordance with the recommendations of the PRISMA (Preferred Reporting Items for Systematic Review and Meta-Analysis) statement [17]. The search of the scientific literature was conducted between October and November 2022 in the following electronic databases: PubMed, SPORTDiscus, Web of Science, and ScienceDirect. Keyword and category search terms were used as follows: (1) the first search category was related to terms associated with RWL, such as (“Dehydration” OR “Rehydration” OR “water-electrolyte balance” OR “water deprivation” OR “water loss” OR “Acute weight loss” OR “body weight loss” OR “Weigh-in” OR “Weight Cutting”); (2) the second category was related to CS, with the terms (“combat sports” OR “fighting sports” OR “striking sports” OR “boxing” OR “wrestling” OR “karate” OR “taekwondo” OR “judo”); while the third category (3) referred to athletic performance (“performance” OR “physical condition*” OR “recovery”). Categories (1)–(3) were related to each other with the Boolean operator “AND”. 

### 2.2. Inclusion and Exclusion Criteria 

For the article selection in the present review, inclusion criteria were previously established as follows: (1) The subjects had to be competitors in CS with at least >3 years of competitive experience and carrying out RWL strategies in order to fit into a lower category than their natural weight. (2) The studies had to take into consideration the effects of the weight loss strategies on athletic performance parameters (at least two measurement points, that is, normal conditions and dehydration/weight loss condition). (3) The subjects had to participate in a real competition or in at least a simulation with comparable conditions or effort. (4) Original research articles were written in English or Spanish and are available in full text. 

### 2.3. Study Selection

The complete search process was conducted by two independent researchers (M.S.-M. and G.O.-D.) and supervised by a third one (L.M.M.-A.). Both authors independently used the search terms to examine the literature in the different meta-search engines selected, subsequently analysing and screening the titles and abstracts of the resulting articles after removing the duplicates, and using an excel sheet prepared for that purpose. In the event of any discrepancies between both researchers during the inclusion process (initial disagreement rate: 8.3%), there was a discussion and resolution by consensus. In case such discrepancies persisted, the third reviewer moderated the final consensus process on the inclusion of the article in the following phase (L.M.M.-A.). To determine the final reliability between the reviewers after the records screened, Cohen’s Kappa statistic (Κ) was calculated (Κ = 0.93) [18]. The data extracted from the selected articles were as follows: study design, participants (number, gender, age, weight, sport), method of weight loss, magnitude of rapid weight loss and recovery time, duration and time-points assessed, tests and performance parameters, other records or variables analysed, and finally, main results. 

**Figure 1:**
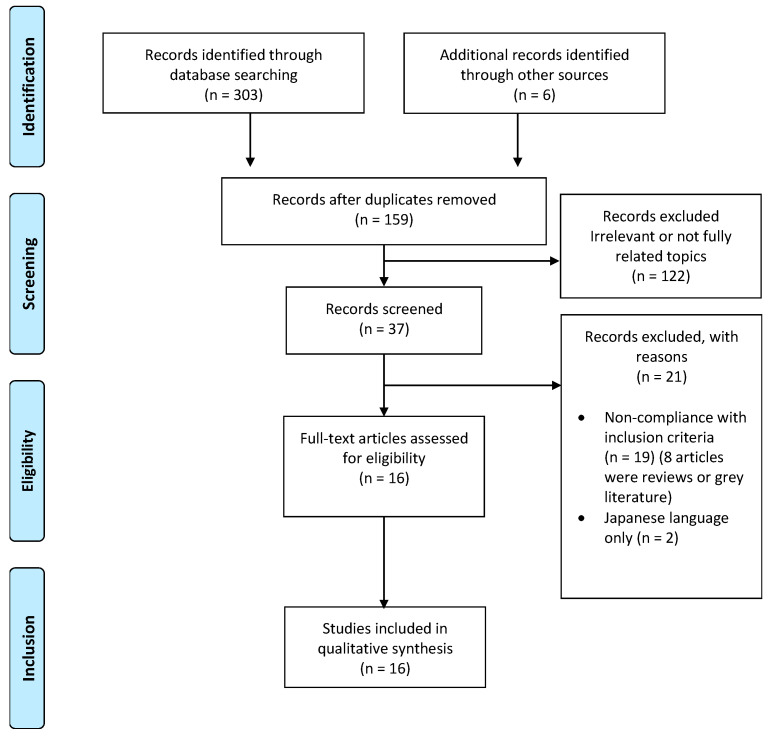
PRISMA flowchart for the systematic review, describing the final selection process of articles included in the qualitative analysis or synthesis.

The article selection process was as follows: A total of 303 studies were identified through the different databases. Six additional articles of interest were identified, as listed in the references section of recent systematic reviews obtained from the initial search conducted [1,2,5]. After eliminating the 150 duplicated articles, a total of 159 articles remained. Following title/abstract review, 122 articles were excluded as they did not meet the inclusion criteria, leaving a total of 35 full text articles assessed for eligibility. Subsequently, 21 were excluded with specific reasons, as 19 of them did not meet the inclusion criteria (8 of them we systematic reviews of grey literature without peer-reviewing process), and 2 were eliminated as they were only available in Japanese. Therefore, 16 studies were finally included in this systematic review. Further details on the identification, screening, eligibility, and inclusion processes can be found in Figure 1.

### 2.4. Assessment of Methodological Quality 

The assessment of methodological quality was also conducted by two independent researchers (M.S.-M. and G.O.-D.), supervised and corrected when necessary by a third one (L.M.M.-A.). For this purpose, the PEDro scale was used [19], containing originally 11 questions or items that can be answered with a “0” or “1”, depending on compliance with criteria, with a maximum of 11 points. In this case, due to the nature of the scientific literature analysed, blinding participants and/or researchers from experimental conditions is quite complicated or not possible. For that reason, the three questions related to blinding process were removed from the original scale as previously made by other authors with similar methodological quality scales [1,20,21]. 

For the acceptance of the articles, the authors created three categories in order to score the quality of the articles: very good–excellent (7–8), moderate–good (5–6) and poor (≤4). 

The scores relative to the assessment of methodological quality for each article are detailed in Table 1. 

All articles responded had a minimum score of 5, favouring their subsequent acceptance and analysis. The results of all the studies were correctly reported, alluding to all the participants, regardless of the group in which they were allocated, and analysing in depth the key results considered by the authors. On the other hand, it should be noted that the allocation of the sample into groups, as well as the data collection by the researchers (items eliminated), were not blinded in any case. Concerning the item “D-Groups were similar at baseline with respect to the most important prognostic indicators”, that is, in those studies without a division into different groups, the item assessment has been omitted.

## 3. Results

### 3.1. Main Characteristics of the Studies 

The characteristics of the different studies are shown in Table 2. All studies included in this review recruited male and female athletes within the intervention groups (*n* = 184, 174 male and 10 female athletes). All subjects were athletes from CS, with a minimum of 3 years of practice, as well as certain experience in RWL strategies.

Same-day weight loss by sweating and fluid deprivation was performed in three studies [22,23,24]. The rest of the included studies provide at least a few days for weight loss, also through calorie, energy and/or fluid restriction, specifically a period of 5 days [25,26,27], 1 complete week/10 days [28,29,30,31,32,33,34], or 3/4 weeks [35,36]. In addition, there was a final study comparing progressive RWL performed weeks in advance (3 weeks of calorie restriction) with those performed faster or at shorter notice (59 h prior to weighting) [37].

In addition to the RWL typologies and their programming over time, tests applied to aerobic and anaerobic capacity such as the Wingate [23,25,35,37] or an arm ergometer [27] stood out as performance analysis methods. Similarly, studies applied specific tests for each combat speciality [24,25,26,31,33,34,36] and measures aimed at determining the athlete’s strength/performance [22,28,29,30,31,32,33,34,36,37] or psychological state [29,30,32,36]. In addition to the tests applied to performance, data collection aimed at controlling body composition and weight [22,25,27,28,29,30,31,32,34,35,36], and the analysis of blood components [22,25,26,27,28,29,30,31,37] and urine [22,23,30,34,35] were of particular importance. 

### 3.2. Studies on RWL with No Effects on Performance-Related Parameters

Directly alluding to the findings from the literature, six studies included in the present review demonstrated that RWL protocols of around 5% of body mass do not produce significant changes in performance parameters (minimum of *p* > 0.5 in all cases). McKenna–Gillum [23] reported that mean lower-body anaerobic power was not significantly different between groups (*p* = 0.877) and time points, (*p* = 0.809), after an intervention of ~3% body mass loss. Their data support that dehydration at this percentage does not impair athletic performance; therefore, in attaining ≤3% of ideal weight before weigh-in, anaerobic power will not be hindered. 

Taking into consideration specific performance parameters within the martial discipline, Artioli et al. [25] revealed no group (control vs. 5% body mass loss), time or interaction main effects for time structure patterns or number of attacks during judo combats. In addition, no main group or interaction effects for mean and peak power and total work were found between groups, as well as no differences and effects for other complementary parameters measured such as haematocrit and plasma lactate. Thus, as suggested by the authors, RWL strategies did not affect athletic performance.

Similarly, Lopes-Silva et al. [26], applying a 5% of RWL, showed no significant main effects on condition (control, placebo, and caffeine groups) for the number of judo throws or special judo fitness test performance index. Concerning the RPE, the group taking caffeine pills during the recovery period showed lower values compared to the placebo group (*p* < 0.05). Conversely, the La- values became elevated in caffeine group in comparison to control or placebo group, increasing as the special judo test progressed (*p* < 0.05). There was no effect of condition for HR values (*p* > 0.05). 

Along the same lines, a RWL of 5% led to no significant changes between conditions in plasma lactate, variables analysed during recovery (energy and macronutrient intake), or performance variables (mean power, peak power, or total work) (*p* > 0.05) [27]. Likewise, Barbas et al. [31] reported no differences for all dependent variables measured and compared between baseline (T1) and pre-1st match (T2) following an isolated weight loss process (~6% BM loss) such as anaerobic- and wrestling-related performance tests, heart rate, lactate concentration, CK, glucose, and other physiological parameters (*p* > 0.05). 

Moreover, RWL is capable of achieving greater weight loss compared to progressive weight loss strategy (*p* < 0.01) [37]; however, no differences were found for sprint performance—30 m—and total work output during the Wingate test after a 5% of body weight loss following RWL strategy. On the contrary, their results suggested a better mechanical functioning after a progressive weight loss strategy (*p* < 0.01), even if the RWL has preserved capability of the neuromuscular system to produce force. In any case, the RWL reported no general impairments on performance-related parameters. 

### 3.3. Studies on Negative Effects of RWL on Performance-Related Parameters

Ten studies included in this review reported negative effects or impairments in performance and physiological parameters due to RWL strategies. 

Kraemer et al. [28] showed a progressive loss of performance when data were collected during a 2-day within a five-bout wrestling tournament (~6% body weight loss). Significant differences (*p* < 0.5) were found in the perceived fatigue of the wrestlers after each bout compared to the initial value (1.21 vs. 6.75 match 1; 1.8 vs. 6.8 match 2; 2.8 vs. 6.92 match 3; 2.38 vs. 7.33 match 4; 2.54 vs. 7.58 match 5). In agreement with the consistent increase in fatigue values over the tournament, there were significant lower values in the handgrip and “bear hug” tests (*p* ≤ 0.5), especially in the first fight, together with reductions in knee flexion, fast knee extension, and slow elbow extension torque values (isokinetic strength impairments) as the tournament progressed (*p* ≤ 0.5). 

Physiological changes were observed as well, specifically a chronic reduction in resting serum testosterone, a general low testosterone response, and an increase in dopamine concentration throughout the tournament. Other hormonal parameters and blood components were modified as well, showing very elevated values compared to baseline and pre-fight ones (i.e., plasma osmolality, creatine kinase, and cortisol).

Furthermore, significant differences in several physical and physiological parameters after a 3-h dehydration period (3–4% body weight loss) were found by Barley et al. [22]. Differences were revealed in the ability to perform repetitions in isokinetic strength tests after the dehydration period, with a reduction in the number of contractions completed during the fatiguing exercise protocol (17 ± 7 vs. 23 ± 8, *p* < 0.001), and an increase in perceived fatigue after dehydration (17 ± 6 vs. 6 ± 2, *p* < 0.003) and in the following 3 h (11 ± 5 vs. 6 ± 2, *p* < 0.012). In addition, significant variations right after dehydration were found in the body composition of the wrestlers (Pre 80 ± 10 vs. Post 77 ± 10, *p* < 0.001, for dehydration group), as well as serum osmolality (293 vs. 285.1 mOsm, *p =* 0.003); hematocrit (45 vs. 43, *p =* 0.034), resting heart rate (117 ± 21 vs. 56 ± 9, *p* < 0.001), and tympanic-core temperature (*p* < 0.001) for dehydration vs. control group, respectively. Significant and negative differences in urine osmolality and urine specific gravity were shown in dehydration group compared to the control group at the end of 3 h of dehydration and were maintained after the 3 h rehydration period (*p* < 0.001). 

In a comparable way, Kurylas et al. [35] showed significant impairments of anaerobic performance parameters after 4 weeks of energy and fluid restriction (~6% BM loss). After analysing the values of a Wingate test, decreases in peak power (*p* = 0.021), mean power (*p* = 0.039), and total work performed (*p* = 0.036) were shown comparing a 4-weeks period and the day before the fight (weigh-in), as well as weigh-in and the fight day after the hydration. A significant increase in urine osmolality (*p* < 0.05) and urine specific gravity (*p* < 0.01) were also noted in a comparison between 4 weeks before and the day before the fight. Finally, it is noted that the largest statistical difference found between the data collection 4 weeks prior to the fight and the pre-fight date is based on changes in lactate concentration and the body mass in the wrestlers.

Applying a (~5% BM loss), Degoutte et al. [30] reported reduction in several variables, such as left handgrip (53.6 ± 2.7 vs. 50.4 ± 2.5 kg; *p* < 0.01, −5%) or testosterone and insulin concentrations, as well as an increase in ACTH and cortisol, among other variables (*p* < 0.05). The experimental group had several changes concerning the biochemical test, with a significative increase in uric acid, glycerol, and free fat acid concentrations (*p* < 0.05).

Furthermore, even if Koral–Dosseville [36] found no differences in some variables measured between baseline and weight-in time point (i.e., squat jump and countermovement jump ratio, or repetitions of favourite judo movements for 5 s), a procedure main effect of *p* < 0.01 and a group x procedure interaction of *p* < 0.5 were reported within the specific judo test for 30 s after the RWL period (2–6% BM loss). 

Additionally, taking into consideration a secondary analysis, they found differences between genders, where male athletes reported higher values compared to female competitors for squat jump, countermovement jump, and mean power. Likewise, they reported significant negative changes for psychology-related variables. Specifically, for the experimental group (2–6% BM loss), the POMS scores were increased for tension (only female) and confusion (both genders) (*p* < 0.05) and decreased for vigour in both genders (*p* < 0.05).

Similar findings were revealed by Filaire et al. [29], where a significant increase for the states of confusion, anger, fatigue, and tension, as well as a decreased level of vigour, were found after the weight loss period (~5%). A significant decrease in left handgrip values (*p* < 0.05) and a higher level of triglycerides and free fatty acid 24 h before the championship (*p* < 0.05) were also found. 

Lastly, Barley et al. [22] analysed other psychological parameters in their study, such as perception of tension, depression, confusion, and anger (POMS) being greater after dehydration when compared with control (*p* < 0.5). Degoutte et al. [30] also found an increased score for tension, fatigue, and anger, while vigour decreased in a significant way. 

Including a large percentage of body mass loss, two studies were included in this review. Alves et al. [34], with a 9.9% BM loss, demonstrated an important decline in handgrip strength from baseline to weigh-in (*p* = 0.001) and match time (*p* = 0.001), and a significant increase in urine density values from baseline to weigh-in and match time (*p =* 0.05). In the same line, Camarço et al. [32] presented a two-case study with athletes carrying out a 9.1 or 5.3% BM loss strategy, showing an important decrease of 55–45.1%, respectively, in salivary nitrite concentration during the weigh-in after RWL. This percentage was only partially recovered through the replacement (35.9–25.5% for 9.1 and 5.3% of BM loss). Concerning cognitive tasks, the number of mistakes at any time was higher with 9.1% compared to another case study with a lower percentage (5.3%), especially during the fight day. Muscle power decreased at weigh-in time point in both cases, not being re-established after the weight gain (only the athlete with 9.1% of BM loss). 

Moreover, Moghaddami et al. [24] found a reduction in the performance after a period of 3 × 20 min in a sauna (~4.3% BM loss). The analysis of kinematic parameters revealed an acute dehydration impact and significant variations in almost all body segments (shoulder, pelvis, knee, trunk, and thigh) concerning linear and angular VelMax, and position (*p* < 0.001). Additionally, significant reductions in body composition parameters were shown (weight pre, 71.1 ± 11.8 vs. post, 68.0 ± 11.4, *p* = 0.001; %fat pre, 4.9 ± 1.4 vs. post, 4.8 ± 1.35, *p* = 0.008; total body water pre, 45.9 ± 6.4 vs. post, 43.2 ± 6.0, *p* = 0.001).

Finally, Liu et al. [33], also from a biomechanical point of view, found that even if the total reaction (central nervous system) is better thanks to the replacement period, a complete weight cutting process (including a ~4.5% BM loss and a replacement strategy) has a negative effect on the peripherical nervous system reaction time (limbs’ movements) being −19.23% slower than normal, as well as a negative effect on striking accuracy (−7.46%). Three striking styles were also negatively affected in terms of power generation (−10 to −63%).

## 4. Discussion

This systematic review focuses on the effect of different RWL strategies in athletic-performance-related parameters of combat sports in real or simulated competitive environments. The debate over whether or not such strategies affect athletic performance remains unanswered in a certain way, since multiple factors come into play, such as the different strategies used for weight loss, the percentage of body weight lost, and the orientation towards either dehydration or food/calorie restriction [1]. Moreover, the body’s compensatory effects following severe or very severe restriction result in a “rebound” reaction and subsequent weight gain of equal or greater magnitude than the restriction, which competitors should monitor and be aware of [38,39]. 

In addition, one of the key elements in weight loss strategies is to know where the limit of weight cutting lies. The majority of existing negative evidence is related to extreme body weight loss between 10 and 17% [40,41]. The debate on the extreme weight cut appears to divide opinion within the combat sports community (coaches, competitors, fans, and so on). It remains complicated to establish what is the limit of weight cutting and, at least, what are the real effects of weight cutting on performance. The weight cutting can change in severity, finding some competitors just dropping or adjusting their weight just a few kilograms or pounds, while other fighters develop extreme weight cutting strategies, losing more than 11–12% of body mass in trying to obtain a clear advantage over their opponents [42]. 

According to the studies analysed in this review, the percentage of body weight that can be lost without jeopardising athletes’ performance in a significant way could be set between 3% and <5% in general terms. The main problem encountered is the diversity in study designs, as well as the percentages and weight loss strategies used in the investigations. Actually, this is a critical interval since previous data have established somewhat contradictory statements, where some studies indicate that drastic weight reduction affects performance, while others reveal no negative impact on it, at least up to a certain percentage of body weight loss. 

On the one hand, studies by Artioli et al. [25] and Lopes-Silva et al. [26] showed in a sample of professional judo fighters that a loss of up to 5% of body weight, followed by 4 h of recovery, did not affect competitive performance. Along the same lines, the studies by Mendes et al. [27] and Fogelholm et al. [37] provided evidence that RWL, as long as it is less than 5% of body weight and has a few hours to refeed and rehydrate after the weigh-in, does not affect high-intensity performance, sprint performance, and anaerobic power, at least in male athletes.

This is supported by studies where no significant changes were found in muscular activity even the athletes performed a 4% hypohydration [43,44]. Additionally, Cengiz [45] and Barbas et al. [31] already established that body mass loss should exceed 5–6% before impairments in anaerobic and specific performance-related parameters appear. 

The above studies maintain a specific limit of maximum weight loss to ensure control of performance loss, but there are studies that make specific reference to fluid loss, making a comparison only between a baseline state of hydration and a weight adjustment using dehydration-only strategies. In this regard, Yamashita et al. [46] demonstrated that a hypohydration level of 2.25% body mass did not affect high-intensity intermittent exercise performance compared to baseline values, with no effect on the various lactic and alactic mechanisms of competitors. Along the same lines, McKenna–Gillum [23], established the limit of weight loss due to dehydration at 3%, agreeing with the risks associated with the statements of Yamashita et al. [46]. They revealed that dehydration at ~3% body mass loss did not impair anaerobic power. Therefore, a wrestler attempting to adjust the weight in order to compete in a different category should be focused on ≤3% BM loss before weigh-in.

However, Yamashita et al. [46] stated that the conditioning factor for not exceeding the limit of approximately 2–3% hypohydration lies in the affection of the aerobic system through this fluid restriction, which can influence the energy supply to the athletes, conditioning the maintenance of anaerobic exercise during intermittent exercise, and subsequently, the athletic performance. Thus, aerobic performance is one of the keys to competitive performance in CS [47], so controlling the factors that influence aerobic performance is essential in the training processes of these disciplines. In this regard, it is important to remember that time-motion analysis in combat sports have reported an important ratio of high-intensity efforts, being a critical factor within the match performance, especially when prolonging combat during the bout (aerobic metabolism will be required) [48]. 

On the other hand, Brito et al. [49], stated that a >3% body mass loss for a period of less than 48 h affects the competitors’ ability to produce force and the ability to develop speed. Even if weight loss over several days (5–12) through fluid/food restriction could not cause performance alterations, a higher percentage of body mass loss could be risky for the maintenance of athletic performance. Actually, there are studies showing a negative impact of RWL to anaerobic capacity, maximal strength, power, and high intensity effort repeated over time up to 24 h after the weight loss [34,50,51,52,53]. In this regard, combat sports with higher competition length are more prone to present a real risk of performance alterations derived from RWL strategies. 

Furthermore, Barley et al. [22] agreed in their study with the establishment of 3–4% as the limit of weight loss due to dehydration, finding significant differences in physical (isokinetic strength, perceived fatigue) and physiological parameters (body composition, resting heart rate, urine and serum osmolality, among others) after a dehydration period. Moreover, other studies give support to this percentage interval, demonstrating alterations and decreased values in physical and physiological parameters when applying a higher %BM loss (~5% or ≥9%), such as handgrip strength and muscle power output [29,30,32,33,34], specific sport discipline test [36], testosterone and insulin levels [30], as well as alterations in concentration/level of cortisol, uric acid, and urine density, among other biochemical parameters [29,30,32,34]. 

In this line, Kurylas et al. [35] demonstrated significant impairments in anaerobic performance when applying a 29-day hypohydration period (~6% body mass loss), showing lower values in peak and mean power, as well as total work performed (anaerobic capacity). This could be related to the increment in central temperature, affecting the muscle strength production sequence. This is mainly through the reduction in motor cortex activation and the development of peripheral stimulation, in addition to the aforementioned decrease in power output [54,55]. 

Within the athletic performance, it is also worth mentioning the influence of RWL strategies (~4 to ≥6% body mass loss) on psychological aspects. Barley et al. [22] revealed that a greater loss would lead to cognitive fatigue, directly affecting the performance of the athletes, and could be maintained over time even after fluid replenishment. In addition, Barley et al. reported higher scores in perceptions of tension, depression, confusion, and anger after the dehydration period compared to control group. Similarly, Koral–Dosseville [36], Filaire et al. [29] and Degoutte et al. [30], reported altered scores in the profile of mood states dimensions; specifically, a lower score in vigour and increased scores for tension, fatigue, depression, confusion, and anger were reported. 

These statements coincide with the data extracted from the reviews by Lakicevic et al. [5], Ceylan et al. [6], and the study by De Sousa Fortes et al. [9]. In those studies, a large weight loss in a short period of time, produced negative effects on psychological aspects and moods, together with a decrease in metacognitive performance and the possibility of attenuating attention and anticipation skills, thus affecting decision-making. In the same line, Camarço et al. [32] showed significant changes in cognitive tasks, where the number of mistakes within the tests at any time point was higher in the subject with 9.1% of body weight loss. 

Continuing the line of RWL strategies studied through dehydration, the study by Moghaddami et al. [24], added an element of great importance, which is that acute fluid loss through dry sauna has negative effects on biomechanical parameters, linear and angular maximal velocity, and joint position in elite wrestlers. The maintenance of specific techniques in professional wrestlers is as important as their strength and endurance capacity [56], so ensuring the ability to maintain movements over time is essential, and weight loss strategies cannot condition their execution under any circumstances. Under a similar biomechanical perspective, Liu et al. [33] found that the weight cutting had negative effects on peripheral nervous system reaction time (concerning the specific limb’s actions) and accuracy. 

Together with the RWL, the present study provides evidence that, according to the existing scientific literature, a progressive weight loss that combines food and hydration control strategies does not have a negative influence on performance, something that is caused by short-term or very short-term weight losses with a more strenuous percentage of body mass loss [36,37]. Based on this, the contribution of the study by Matias et al. [57] should be noted, where it was stated that magnesium supplementation during weight loss protocols favoured the maintenance of athletes’ performance, as its loss through sweating is very high and its intake is usually insufficient. Magnesium supplementation has been shown to be very common in elite sports and of great value for the maintenance of muscle capacity in athletes, so its application to the world of CS is amply justified [58].

Related to the importance of weight loss strategies is the relevance of recovery from weight loss. The recovery period between weight control and competition is usually 24 h or less, which seems insufficient to regain hydration status, as it affects athletes’ power values compared to their pre-restriction levels [28,35,59]. Indeed, Petterson–Ekström [60] could not restore the hydration process completely for the 42% of the studied population, classified as “severely dehydrated”. Moreover, Barbas et al. [21] applied a recovery period of 12 h, where the wrestlers regained only 1.2% body weight. In the same line, Alves et al. [34], Carmaço et al. [32], and Kurylas et al. [35] (24 h in all cases), attempted to fully rehydrate the competitors without success. Consequently, this information reveals that a period >24 to 48 h is necessary in order to complete an adequate rehydration process after a short-term weight cycling. 

However, Fogelholm et al. [37], stated that a period of between 12 and 14 h is enough for the athlete to recover after a period of rapid restriction of 59 h, likely demonstrating that weight losses in very short periods of time can in turn be recovered in very short periods of time as well. 

That being said, as mentioned above, these kind of strategies can clearly be more detrimental than more progressive ones [2]. Actually, a very fast (>1 kg·d^−1^) and/or very aggressive rehydration (>6%) could provoke performance impairments [37]. Nor should it be forgotten that calorie restriction and dehydration are different concepts or strategies that should not be confused, as well as the feeding back and rehydration processes. 

Interestingly, there are competitions lasting more than 1 day, so rehydration process, weight maintenance, and the accumulation of bouts across the rounds could negatively affect athletic performance. Kraemer et al. [28], in their study applied to wrestling, stated that there are physiological and performance decreases due to RWL, and their impact is progressive over 2 days of competition. However, such a claim should be approached with caution, as the tolerance of CS athletes to weight loss strategies is very high; thus, the changes that occur could be due to the concomitant accumulation of other stressors (the competition itself, psychological stress, and the accumulation of bouts during competition) [28,31,61].

In any case, it should be recalled that the compensation period after the RWL is the time when athletes can refuel and rehydrate, and the duration of this recovery period is decisive for the performance in subsequent bouts [13]. This time margin will depend on the type of event organisation, and there may be a weigh-in prior to the day of the competition or within the competition day itself, as well as variations in the order of bouts allowing the athletes a greater or lesser margin for recovery. Concerning this differentiation, the average number of hours available to competitors in amateur events is usually between 2.5 and 5 h in the same session/day, being considered insufficient time for the replenishment of food and hydration. Although extreme methods may prevent a loss of athletic performance, they may result in an increased tendency to engage in potentially harmful RWL procedures [62].

Despite the limited number of hours available in amateur competitions, it has been generally shown that complete rehydration and muscle glycogen replenishment require at least 48 h, so the common time between weigh-in and competition is not considered to be enough for the recovery and optimal performance of the athlete [10]. However, as Tarnopolsky et al. [63] have already pointed out, the possibility of achieving partial rehydration seems possible, achieving improvements in athletic performance (or less negative effects) in an average replacement time of 17 h. 

In any case, some authors [64] suggest that observed windows of 32 h for recovery could promote aggressive “weight-making” strategies, involving a clear risk to an athlete’s well-being. 

Lastly, the scientific literature shows that RWL methodologies are more effective and behave differently in male compared to female athletes, which should be taken into consideration among athletes and coaches [7]. Koral–Dosseville [36] found differences between genders after a RWL strategy intervention, where higher values for squat jump, countermovement jump, and mean power were found in male athletes. However, male competitors had greater losses in countermovement jump, mean power, and the specific judo test for 30 s. 

In this context, it should be pointed out that the number of female competitors in combat sports has increased considerably in the last few years, and there has been a growing interest in the research community. In fact, studies in other sports disciplines or contexts highlight the importance of analysing the relationship between the practice of this type of weight loss strategies and physical and physiological performance parameters, especially when analysed together with the menstrual cycle as a variable to consider [65,66]. Last but not least, the scientific literature argues for more gender balance in sports science research.

## 5. Conclusions

After analysing the studies on the effects of RWL in CS on the athletic performance from physical, physiological, and psychological perspectives, the current systematic review can be assured that the most advisable RWL methodology to guarantee the athletic performance of the competitor in general terms should not exceed 3% to ≤5% of total body weight loss, followed by ≥24 h hours for rehydration after the weigh-in in order to have an adequate recovery (at least partially, since the common timing between weigh-in and the competition is ~1 day). This could be within acceptable limits for competition, assuming no or minimal decrease in athletic performance, and probably being compensated by the competitive advantage of fighting in a lower weight category.

Concerning the time needed to perform such RWL, a progressive weight loss over several weeks is recommended, being even more relevant in competitions lasting several days and involving multiple bouts or qualifying rounds. On the other hand, the big question remains whether the benefit of downgrading and competing against a “smaller” opponent outweighs the costs and risks of a possible reduction in athletic performance due to drastic RWL strategies.

Despite all this and its widespread use among CS athletes, it is important to highlight the need to raise awareness among coaches about avoiding these types of weight control programmes in youth athletes, as they could have a negative effect on their development and affect their hormonal balance [38].

Finally, it is important to consider the possible limitations found in this research. The most relevant is the implementation of almost all the programmes only in male athletes (15 out of 16 studies), whereby the results obtained may differ from those applicable to female wrestlers. In addition, no ethnic identity was reported in the selected studies, which could be a parameter to take into consideration in new original studies when implementing this type of strategies.

## Figures and Tables

**Table 1:** Results obtained in the evaluation of study quality.

Studies	A	B	C	D	E	F	G	H	Score
Barley et al. (2018) [22]	1	1	0	1	1	1	1	1	7
McKenna-Gillum (2017) [23]	0	1	0	1	1	1	1	1	6
Moghaddami et al. (2018) [24]	1	0	0	--	1	1	1	1	5
Artioli et al. (2010) [25]	1	0	0	1	1	1	1	1	6
Lopes-Silva et al. (2014) [26]	1	1	1	--	1	1	1	1	7
Mendes et al. (2013) [27]	1	0	0	1	1	1	1	1	6
Kraemer et al. (2001) [28]	1	0	0	--	1	1	1	1	5
Filaire et al. (2001) [29]	1	0	0	--	1	1	1	1	5
Degoutte et al. (2006) [30]	1	1	0	1	1	1	1	1	7
Barbas et al. (2011) [31]	1	0	0	--	1	1	1	1	5
Camarço et al. (2016) [32]	1	0	0	--	1	1	1	1	5
Liu et al. (2022) [33]	1	0	0	--	1	1	1	1	5
Alves et al. (2018) [34]	1	0	0	--	1	1	1	1	5
Kurylas et al. (2019) [35]	1	0	0	--	1	1	1	1	5
Koral-Dosseville (2009) [36]	1	0	0	1	1	1	1	1	6
Fogelholm et al. (1993) [37]	1	0	0	1	1	1	1	1	6

A—Choice criteria were specified; B—Subjects were randomly assigned to groups (in a crossover study, subjects were randomly distributed as they received treatments); C—Assignment was concealed; D—Groups were similar at baseline with respect to the most important prognostic indicators; E—Measures of at least one of the key outcomes were obtained from more than 85% of the subjects initially assigned to groups; F—Results were presented for all subjects who received treatment or were assigned to the control group, or when this could not be completed, data for at least one key outcome were analysed by “intention-to-treat”; G—Results of statistical comparisons between groups/points were reported for at least one key outcome; H—The study provides point and variability measures for at least one key outcome.

**Table 2:** Characteristics and main outcomes of the studies.

Study	Design or Type of Study	Subjects	Sport	Weight	Weight Loss Method	RWL (% of BM Loss)	T. Rec	Time-Points Assessed	Performance Parameters	Other Registers	Main Outcomes/Conclusions
Barley et al. (2018) [22] (Australia)	ED	14 M (25 years ± 4 years)	Combat sports (>2 years of experience)	80 ± 11 kg	Passive heat exposure (3 h)	3–4%	3 h	T0: familiarisation T1: experimental 1 (7 days later) T2: experimental 2 (at least 7 days later)	Mood, cognitive function, neuromuscular function (isometric knee extension—MVIC), vastus lateralis and medialis EMG.	BM, BP, CT, TT, HR, blood, urine	Acute dehydration of 3.2% followed by 3 h of recovery impairs muscle strength-endurance and increases the perception of fatigue, with no change in central and peripheral function markers.
McKenna–Gillum (2017) [23] (United States)	ED	7 M (19.75 ± 1.67 years)	Wrestling	76.8 ± 4.32 kg (11.6 ± 4.32% fat)	Running at 70% VO_2_max (heating 30 ºC)	~3%	1 h	T1: euhydration (pre-exercise) T2: in 3% loss (post-exercise) T3: rehydrated (1 h after) through water or glycerol (3%)	Anaerobic power: Wingate test (Pm) with 30 sec. at maximum intensity	Urine specific gravity, OsmSal	No significant changes in performance and complementary physiological parameters were found.
Moghaddami et al. (2018) [24] (Turkey)	CuaD	13 M (18.38 ± 1.32 years)	Wrestling (8 years of experience)	71.11 ± 11.80 kg	By sauna (3 × 20 min at 60–70 °C, 5 min. rest intervals)	~3.5–4%.	18 h	T1: pre-test (without dehydration) T2: dehydration ~4.3% T3: 18 h after rehydration (~1.3%)	One-legged knockdowns (three-dimensional, linear and angular analysis)	N/A	Weight loss could affect fighters’ abilities. Negative effects on shoulders, pelvis, and knees joints during a single takedown technique (linear and angular VelMax, and position).
Artioli et al. (2010) [25] (Brazil)	CuaD	14 M EG = 7 (20 ± 4 years) CG = 7 (22 ± 4 years)	Judo (EG = 12 ± 4 years of experience; CG = 13 ± 3 years of experience)	EG = 77.9 ± 12.2 kg CG = 67.3 ± 5.8 kg	5 days for weight loss through their usual methods, food record last 3 days	5%	4 h	T1: pre-loss (5–7 days before) T2: after recovery (4 h)	Multitask Judo specific protocol: 3-maximal *uchi-komi* exercise, combat and Wingate test of 30 s (upper-body).	BC, Glu, La-	Rapid weight loss did not affect performance in experienced judokas when they had ~5% BM loss and 4 h to recover.
Lopes-Silva et al. (2014) [26] (Brazil)	CD (double-blinded, counterbalanced, crossover)	6 M (25.3 ± 5.7 years)	Judo (14.4 ± 8.9 years of experience)	71.1 ± 13.5 kg	Two cycles of 5-day, as they would do in a real competition	5.5 ± 3.4%	4 h (at 3 h caffeine or placebo were implemented)	T1: two cycles of 5-day period (baseline and RWL process) T2: 4 h after weighing (after rec) A 15-day, wash-out period was applied between cycles	3 sets of SFJT with 5 min recovery time (pre baseline or first 5-day cycle; pre RWL application; and post RWL)	BC, RPE, La-, HR	An average of 5% reduction did not affect performance. Caffeine does not increase performance, but decreases RPE and increases plasma lactate concentration compared to placebo.
Mendes et al. (2013) [27] (Brazil)	CuaD	18 M EG = 10 (weight cyclers) CG = 8 (non-weight cyclers)	Judo, Brazilian jiu-jitsu, wrestling, MMA (EG = 12 ± 3 years of experience; CG = 9 ± 3 years of experience)	EG = 77.7 ± 12.3 kg CG = 73.8 ± 9.5 kg	Food diary 3 days before T2, but no fluid records	5% in 5 days	4 h	T0: familiarisation 7 days before T1 T1: 5 days before combat T2: combat day	High-intensity intermittent exercise capacity: ergometer arm (8 × 15 s/20 s passive rec)	BC, La-	No significant changes in performance parameters were found with ~5% BM loss and ≥4 h recovery period
Kraemer et al. (2001) [28] (United States)	CuaD	12 M (19.33 ± 1.16 years)	Wrestling (national and international competitors)	75.33 ± 2.54 kg (7.31 ± 0.72% fat)	1 week (Fluid and food restriction)	~6% (4,6–6.7%) within one week	12 h (before fight 1)	T1: morning baseline T2: evening baseline T3: before and after each combat (2-day, 5-bout wrestling tournament)	Power (upper and lower body), isokinetic strength	BC, T, HR, Treac, Fatigue and biochemical parameters (Glu, insulin, La-, dopamine, plasma vol., Ts, OsmSe, CK, cortisol)	Weight loss in isolation did not seem to affect performance. Resting serum testosterone, lower body power and torque, and upper body strength decreased significantly as the tournament progressed. Perceived fatigue increased during the event.
Filaire et al. (2001) [29] [France]	CuaD	11 athletes (gender and age not specified)	Judo (regional and national level; 10 ± 3.2 years of experience; 6–9 h training per week; 2nd–3rd dan BB)	75.1 ± 2.6 (Category under 73 kg)	7 days of food restriction	4.9 ± 1.2	N/A (no rehydration period taken into consideration)	T1: Period of maintenance T2: After a 7 d food restriction, 24 before a national championship	Physical: Handgrip, vertical jump (SJ-CMJ)	BC, nutritional status and dietary intake, biochemical parameters (Cholesterol, TG and Ph), and the profile of the mood states (POMS)	A 7-day food restriction (~5% of BM loss) negatively affects psycho-physiological and performance parameters. An inadequate intake of carbohydrates and micronutrients could play a key role.
Degoutte et al. (2006) [30] [France]	ED	20 M (age not specified) 10 EG and 10 CG	Judo (national level; ~15 years of experience; 9 h training per week; 1st-4th dan BB)	75.9 ± 3.1 (group A); 73.3 ± 6.3 (group B); Category under 81 kg	1 week weight loss period (Energy and fluid restriction)	~5%	The same morning of the competition (not specified time)	T1: Period of maintenance T2: After a 7 d food restriction T3: 10 min. after the simulated competition	Physical: Handgrip, 30-s horizontal isometric rowing	Anthropometry, dietary intake, biochemical parameters (TG, Glu, alkali reserve, Uric acid, urea, creatinine, insulin, Ts, cortisol, ACTH) and the profile of the mood states (POMS)	Combination of energy restriction and intense exercise training negatively affects athletes physiology and psychology, impairing physical performance before competing.
Barbas et al. (2011) [31] (Greece)	CuaD	12 M (22.1 ± 1.3 years)	Wrestling at international level (12.1 ± 2.9 years of experience)	Weight 72.1 ± 3.6 kg; Body fat 7.6 ± 0.9% (category under 66 kg)	1 week for weight loss through restriction of food and fluid intake, cardiovascular exercise, rope jumping and sauna)	~6% (5.1 to 6.5)	12 h (regained 1.2% body weight)	T1: morning baseline T2: before the 1st match T3: pre and post the 2nd, 3rd, 4th and 5th match	Anaerobic (vertical jumping, hip-back strength) and wrestling-related (bear-hug testing, hand-grip testing)	BC, VO2max, La-, Glu, Fatigue, Hormones, metabolites, HR, CK activity, DOMS, ROM, C-reactive protein, leukocytes, IL-6, oxidative stress	An isolated weight loss process prior to a match did not affect performance and/or inflammatory responses in wrestlers. However, a sustained increasing of muscle damage markers through the championship occurred.
Camarço et al. (2016) [32] (Brazil)	Case study	2 M (22 ± 0 years) first one non-supervised and second one supervised by nutritionist	MMA athletes (3 years of experience)	79.0–75.5 kg, respectively (lightweight class)	1-week RWL before weigh-in (through diet, exercise, water and/or sodium restriction, and exercise with plastic clothes or sauna	Athlete 1: 9.1% Athlete 2: 5.3%	1 day before competing	T1: 5 days before competition T2: 36 h before competition T3: simulated competition day	Handgrip, upper limbs power (chest press exercise on smith machine), and lower limbs power (vertical jumps)	BC, Salivary nitrite concentration, and cognitive test (Stroop Colour-Word Test)	Reduction in salivary nitrite concentration (weigh-in) with non-complete replacement after recovery period. Increased rate of mistakes in cognitive test in athlete with higher%RAWL. Muscular power output decreased (weigh-in), being higher and non-re-established in athlete with 9.1% RWL.
Liu et al. (2022) [33] [China-Canada]	CuaD	7 M (25.7 ± 3.9 years)	MMA athletes (regional level; 5.1 ± 3.0 years of training, and 2.9 ± 2.2 years of competition)	78.8 ± 9.9 kg	Up to seven days (preferred methods and timing for BM reduction and regain)	4.5 ± 1.3%	24 h (5.4 ± 1.4%)	T1: pre-test (baseline, before RWL) T2: post-test (after a 24 h period of rehydration and re-feeding)	Neural response/reaction time, striking mean and peak power and striking accuracy	N/A	A complete weight cutting process shows a positive effect on CNSRT and total reaction. On the contrary, a negative effect on PNSRT (limb’s movements) and accuracy was observed. The power output of three specific strikes styles decreased significantly.
Alves et al. (2018) [34] (Brazil)	CuaD	12 M (20.10 ± 1.20 years)	MMA athletes (4 years of experience)	70.40 ± 1.20 kg	Liquid privation	9.9%	24 h (6.9% regain)	T1: baseline (10 days prior to the official weigh-in) T2: 24 h before competition (weigh-in) T3: minutes before the fight (match time)	handgrip strength, performance (won or lost)	BC and BM, hydration status loss and regain (urine density)	RWL results in a decrease in BM, handgrip strength, and urine density. Despite the rehydration attempt, no variables fully returned to baseline values. Moderate relationship with losing the match.
Kurylas et al. (2019) [35] (Poland)	CuaD	6 M (27.3 ± 0.5 years)	Combat sports (at least 8 years of experience, international level)	79.4 ± 1.1 kg	29 days (Energy and fluid restriction)	~6%	24 h	T1: 4 weeks earlier T2: 2 weeks earlier T3: 1 day before (weigh-in) T4: the fight day (after hydration)	Anaerobic performance: Wingate test (Pp, Pm and Wt)	BM, Hyd, OsmUr	Impairments of anaerobic performance, where despite 24-h rehydration, Pp, Pm, and W_t_-J are still negatively affected. Significant increase in urine osmolality and specific gravity.
Koral–Dosseville (2009) [36] [France]	CuaD	20 (10 M and 10 F; 17.0 ± 1.0 years) EG and CG with 5 M and 5 F each	Judo (national or international level; 9.0 ± 2.4 years of experience; 10–14 h training per week; 1st–2nd dan BB)	70.5–72.3 M 66.7–72.6 F	Energy intake reduction (3 weeks) Sweating with exercise using plastic suits (1 week)	CG = 0–2% BM loss (maintenance) EG = 2–6% BM loss	N/A (no rehydration period taken into consideration)	T1: 4 weeks before the national championship T2: The day before national championship	Vertical jumps (SJ, CMJ), repetitions of favourite Judo movements, 10-rowing moves at 70% RM, power development	BM,% body fat, and the profile of the mood states (POMS)	In general terms, it seems that a combined BM loss procedure has no impact on short-time performance parameters or elastic muscle properties. Some impairments concerning specific judo test, prolonged physical performance, and psychological state.
Fogelholm et al. (1993) [37] (Finland)	CuaD	10 M (mean age 21.6, range 17–31)	Wrestling (*n* = 7) and judo (*n* = 3). National or international level (5–10 years of competitive experience or more)	Average weight 73.4 kg (55.1–93.0 kg)	1st gradual (dietary restriction 3 weeks) 2nd fast (59 h)	5%	5 h loading period in the 2nd RWL procedure	2 weight reductions separated by two months: 1°. 3-weeks by dietary restriction T1: before weight reduction T2: 72 h after 2°. RWL in 59 h T1: At the end of weight reduction (59 h) T2: after rehydration (64 h)	Sprint 30 m, vertical jump and anaerobic test (Wingate test)	Blood chemistry and nutritional status assessment (concentration of vitamins, minerals)	An amount of 5% of body weight loss by any method did not affect the performance of experienced athletes.

Note: ACTH = hormone adrenocorticotropic; BB = black belt; BC = body composition; BM = body mass; BP = blood pressure; CD = crossover design; CG = control group; CK = creatine kinase; CMJ: counter movement jump; CNSRT = central nervous system response time; CT = core temperature; CuaD = Cuasiexperimental design; DOMS = Delayed onset muscle soreness; EG = experimental group; EMG = electromyography; F = female athletes; Glu = blood glucose; h = hours; HR = Heart Rate; Hyd = hydration status; La = plasma lactate concentration; M = male athletes; m = metres; min = minutes; MMA = mixed martial arts.; N/A = not shown; OsmSal = salivary osmolality; OsmSe = serum osmolality; OsmUr = urinary osmolality; Ph = phospholipids; Pm = average power; PNSRT = peripherical nervous system reaction time. Pp = peak power; rec = recovery; ROM = joints’ range of motion; RPE = rating of perceived exertion; RWL = rapid weight loss; s = seconds; SGUr = Urine specific gravity; SFJT = special judo fitness test; SJ: squat jump; T. rec = recovery time between weigh-in and “competition”; ED = experimental design; TG = Triglycerides; Treac = reaction time; Ts = testosterone; TT = tympanic temperature; Vel = speed; VelMax = max speed; Wt-J = total work completed measured in Jules.

## Data Availability

There are no additional data beyond those provided in this article.
